# Regulation of Dual Activity of Ascorbate Peroxidase 1 From *Arabidopsis thaliana* by Conformational Changes and Posttranslational Modifications

**DOI:** 10.3389/fpls.2021.678111

**Published:** 2021-06-14

**Authors:** Shubhpreet Kaur, Prapti Prakash, Dong-Ho Bak, Sung Hyun Hong, Chuloh Cho, Moon-Soo Chung, Jin-Hong Kim, Sungbeom Lee, Hyoung-Woo Bai, Sang Yeol Lee, Byung Yeoup Chung, Seung Sik Lee

**Affiliations:** ^1^Advanced Radiation Technology Institute, Korea Atomic Energy Research Institute, Jeongeup, South Korea; ^2^Department of Radiation Science and Technology, University of Science and Technology, Daejeon, South Korea; ^3^Crop Foundation Research Division, National Institute of Crop Science, RDA, Wanju, South Korea; ^4^Division of Applied Life Science (BK21 PLUS), Plant Molecular Biology and Biotechnology Research Center, Gyeongsang National University, Jinju, South Korea

**Keywords:** abiotic stress, ascorbate peroxidase, chaperone, reactive oxygen and nitrogen species, posttranslational modifications

## Abstract

Ascorbate peroxidase (APX) is an important reactive oxygen species (ROS)-scavenging enzyme, which catalyzes the removal of hydrogen peroxide (H_2_O_2_) to prevent oxidative damage. The peroxidase activity of APX is regulated by posttranslational modifications (PTMs), such as S-nitrosylation, tyrosine nitration, and S-sulfhydration. In addition, it has been recently reported that APX functions as a molecular chaperone, protecting rice against heat stress. In this study, we attempted to identify the various functions of APX in *Arabidopsis* and the effects of PTMs on these functions. Cytosol type APX1 from *Arabidopsis thaliana* (AtAPX1) exists in multimeric forms ranging from dimeric to high-molecular-weight (HMW) complexes. Similar to the rice APX2, AtAPX1 plays a dual role behaving both as a regular peroxidase and a chaperone molecule. The dual activity of AtAPX1 was strongly related to its structural status. The main dimeric form of the AtAPX1 protein showed the highest peroxidase activity, whereas the HMW form exhibited the highest chaperone activity. Moreover, *in vivo* studies indicated that the structure of AtAPX1 was regulated by heat and salt stresses, with both involved in the association and dissociation of complexes, respectively. Additionally, we investigated the effects of S-nitrosylation, S-sulfhydration, and tyrosine nitration on the protein structure and functions using gel analysis and enzymatic activity assays. S-nitrosylation and S-sulfhydration positively regulated the peroxidase activity, whereas tyrosine nitration had a negative impact. However, no effects were observed on the chaperone function and the oligomeric status of AtAPX1. Our results will facilitate the understanding of the role and regulation of APX under abiotic stress and posttranslational modifications.

## Introduction

Plants being sessile in nature are inevitably exposed to various abiotic and biotic stresses (Apel and Hirt, 2004; Mittler et al., 2004). Aerobic metabolism is related to various oxidation reactions that lead to the production of reactive oxygen species (ROS) and reactive nitrogen species (RNS). Stressful environments lead to the overproduction of reactive oxygen/nitrogen species (RONS), with the imbalance created between the production and scavenging causing detrimental effects. However, RONS play an important signaling role in plants and act as key regulators of various metabolic and physiological processes (Mittler et al., 2011; Baxter et al., 2014; Del Rio, 2015). Reactive oxygen species, such as superoxide radical, as well as reactive nitrogen species, such as nitric oxide, interact with each other to yield another type of RONS, peroxynitrite, indicating the cross talk between RONS (Halliwell, 2006; Radi, 2013; Khan et al., 2014). In particular, RONS lead to reversible modifications of various redox sensitive and functional catalytic groups of enzymes through oxidative and nitrosative modifications leading to RONS signaling. S-nitrosylation, S-sulfhydration, S-glutathionylation, and tyrosine nitration are few of the redox modifications caused by RONS. These modifications either reversibly or irreversibly alter the stability, structure, and function of proteins (Begara-Morales et al., 2013, 2014; Radi, 2013; Yang et al., 2015).

Organisms have a vast array of antioxidant systems to combat reactive oxygen species (Halliwell, 2006; Del Rio, 2015). Ascorbate peroxidase (APX) is one of the most important enzymes of the hydrogen peroxide (H_2_O_2_) detoxification system, which prevents the accumulation of toxic levels of ROS by reducing H_2_O_2_ into water using ascorbate as an electron donor (Asada, 1992; Shigeoka et al., 2002). More specifically, APX, which contains different isoenzymes which are present in different subcellular organelles, has been found in various higher plants. Based on their subcellular localization, APXs in *Arabidopsis* are classified as cytosolic (APX1, APX2, and APX6), peroxisomal (APX3, APX4, and APX5), and chloroplastic (stromal APX, thylakoid bound APX), while the intracellular location of another member remains unknown (Kubo et al., 1993; Santos et al., 1996; Panchuk et al., 2002; Chew et al., 2003; Narendra et al., 2006). The expression of the APX1 gene is induced in response to ozone, excessive light, high temperature, oxidative stress, and wounding (Kubo et al., 1995; Karpinski et al., 1997; Storozhenko et al., 1998; Shigeoka et al., 2002; Filiz et al., 2019). Plants overexpressing APX1 showed increased tolerance to salt and temperature stress (Gueta-Dahan et al., 1997; Badawi et al., 2004; Lu et al., 2007). Furthermore, APX1, despite being a cytosolic enzyme, is critical for the protection of chloroplasts from ROS and its absence has been shown to result in the compromise of the photosynthetic machinery (Davletova et al., 2005). In some other studies, *apx1* mutants, deficient in cytosolic *APX1*, were reported to show a sensitive phenotype in response to a combination of heat and drought stress and high-light intensities (Pnueli et al., 2003; Koussevitzky et al., 2008). Similarly, cytosolic APX1 from *Arabidopsis* (*AtAPX1*) was demonstrated to be crucial for tuning the regulation of H_2_O_2_, playing a key role in providing acclimation to a combination of heat and drought stress (Koussevitzky et al., 2008).

As mentioned, APX is classically known for its peroxidase function. However, a recent report on rice APX2 has revealed its additional function as a chaperone molecule. In addition, the conformational changes between the high molecular weight (HMW) and low molecular weight (LMW) forms of the protein have been linked to the dual activity of OsAPX2. For instance, it was shown that the protein showed a structural and functional transition in response to salt stress. The switch from HMW complexes to LMW forms were reported to lead to the dissociation of oligomers, which in turn increased the activity of APX under salt stress conditions (Hong et al., 2018). Previous studies on the peroxiredoxin protein reported the oxidative stress-induced regulation of 2-Cys Prx in human cells and yeast. In particular, 2-Cys Prx, in the oxidized form, undergoes a structural conversion from LMW form to HMW complexes leading to a functional switch from peroxidase to chaperone (König et al., 2002; Jang et al., 2004). Cytosolic ascorbate peroxidase possesses 2 substrate oxidation sites i.e., ascorbate and glutathione, and hence it exhibits dual activity in several plants. A recent report indicated that several plants were separated based on the amino acid composition for the glutathione binding site. Plants displaying an oxidizing ability for both AsA and GSH, such as *Oryza sativa*, were classified under group 1, whereas *Arabidopsis* with only the AsA oxidizing ability was placed in group 3 (Chin et al., 2019). This study focused on the functional and structural status of the dual nature of ascorbate peroxidase in *Arabidopsis thaliana.*

The research on NO and posttranslational modifications (PTMs) have brought in sight many target proteins modified by NO-derived molecules. Proteomics studies have suggested that APX is one of the targets of NO-derived molecules, such as SNO and ONOO^–^. For instance, APX, like many other proteins, is regulated by various posttranslational modifications, such as S-nitrosylation (Begara-Morales et al., 2014; Yang et al., 2015), S-sulfhydration (Aroca et al., 2015), and tyrosine-nitration (Begara-Morales et al., 2016). S-nitrosylation regulates protein function either positively or negatively and promotes oligomerization or redox switch in proteins. S-nitrosylation of NPR1 facilitates oligomerization, which is important for the maintenance of protein homeostasis (Tada et al., 2008). S-nitrosylation of the insulin degrading enzyme (IDE) inhibits the enzymatic activity of IDE and promotes oligomerization (Ralat et al., 2009). Tyrosine nitration can cause lowering of pKa of the hydroxyl group, steric hindrance due to attachment of bulky group, or enhance the hydrophobicity of the proteins. It can result in either gain, loss, or no change of protein functions (Kolbert et al., 2017). In tobacco, tyrosine nitration leads to irreversible inhibition of two major H_2_O_2_ scavenging enzymes such as APX and catalase (Clark et al., 2000). Hydrogen sulfide (H_2_S) is a messenger molecule that is highly reactive and toxic. It is known to modify proteins by reacting with cysteine residues to form persulfide, a process known as S-sulfhydration (Mustafa et al., 2009). S-sulfhydration is known to affect the protein function either positively or negatively, for instance, in *Arabidopsis*, the enzyme activity of APX and glyceraldehyde-3-phosphate dehydrogenase is activated by S-sulfhydration (Aroca et al., 2015). The APX1 from the pea plant is reversibly regulated by S-nitrosylation and irreversibly by tyrosine nitration, creating antagonistic effects (Begara-Morales et al., 2014). Although the effects of PTMs on the peroxidase activity of APX have been studied in *Arabidopsis* and some other plant species, the conformational changes of APX due to PTMs remain unexplored.

In this study, we examined the functional and structural link of the APX1 protein in *Arabidopsis thaliana*, both *in vitro* and *in vivo*. As mentioned, the AtAPX1 protein functions both as peroxidase as well as a chaperone molecule, with this dual function being linked to its structural status. Abiotic stresses, such as heat and salt regulate this dual function and structural status of AtAPX1 through the association and dissociation of APX proteins, respectively. Furthermore, we confirmed the effect of various PTMs on the dual function and structural status of AtAPX1. This work provided a comprehensive study on the effects of abiotic stresses and posttranslational modifications on the dual activity of the APX protein.

## Materials and Methods

### Cloning

The *AtAPX1* gene from *Arabidopsis thaliana* was cloned into the pJET1.2 vector (CloneJET, Thermo Fisher Scientific, Waltham, MA, United States) and further cloned into a pET28a (+) expression vector (Novagen, Madison, WI, United States). The coding region of *AtAPX1* was isolated from the genomic DNA of *Arabidopsis thaliana* by PCR with the gene specific primers having restriction enzyme sites, APX1-*Bam*HI-FP (5′-GGATCCATCCATGACGAAGAACTACCCAACCG-3′) and APX1-*Xho*I-RP (5′-CTCGAGTTAAGCATCAGCAAACCC AAGC-3′), using the *Pfu* DNA polymerase (Solgent, Daejeon, Korea). The amplified DNA fragment was cloned into the pJET1.2 cloning vector and transformed into the DH5α (Promega, Madison, WI, United States) bacterial strain. Then, the DNA fragments were cleaved using the respective restriction enzymes and cloned into the pET-28a vector and further transformed into the BL21 (DE3) (Invitrogen, Carlsbad, CA, United States) bacterial strain. Positive clones were confirmed through DNA sequencing and selected for protein expression.

### Expression and Purification of Recombinant Proteins

The *AtAPX1* gene cloned in pET28a was induced using 0.3 mM isopropyl-β-D-thio-galactopyranoside (IPTG) (Promega, Madison, WI, United States) for 4 h at 37°C. The obtained His-tagged fusion protein was purified using a nickel-nitrilotriacetate-agarose (Ni-NTA) column (Peptron, Daejeon, Korea) following the manufacturer’s instructions with phosphate-buffered saline supplemented with freshly prepared 1 mM ascorbate. The protein was eluted using 50 mM Tris–HCl (pH 7.5) with NaCl and thrombin for cleaving the His-tag at 4°C. Purified proteins were exchanged with 50 mM Tris–HCl (pH 7.5) using Amicon centrifugal columns (10 k NMWL) (Merck Millipore, Burlington, MA, United States) following the manufacturer’s instructions, to be used for biochemical analyses.

### Size Exclusion Chromatography

Size exclusion chromatography (SEC) was performed for further purification and determination of the molecular weight of recombinant AtAPX1 proteins. SEC was carried out using fast protein liquid chromatography (AKTA, Amersham Biosciences, Uppsala, Sweden) with a Superdex 200 10/300 GL gel-filtration column (Amersham Biosciences) following previously described methods, with minor modifications (An et al., 2011). Briefly, 50 mM Tris–HCl (pH 7.5) buffer was used to equilibrate the column and run the protein at a flow rate of 0.5 mL/min at 4°C. Absorbance was monitored at 280 nm. Fractions containing the desired protein were pooled and concentrated using Amicon centrifugal columns (10 k NMWL) (Merck Millipore). The column was calibrated using blue dextran (>2,000 kDa), thyroglobulin (669 kDa), ferritin (440 kDa), aldolase (158 kDa), conalbumin (75 kDa), ovalbumin (44 kDa), and carbonic anhydrase (29 kDa).

### Ascorbate Peroxidase Activity Assay

The ascorbate peroxidase activity of purified protein was determined using ascorbate oxidation as previously described, with slight modifications (Nakano and Asada, 1981). The enzymatic activity of the total recombinant protein and fractions (F1-F3) separated by SEC was monitored by measuring the decrease in absorbance at 290 nm. The activity was measured immediately after adding 1 mM H_2_O_2_ to a 500 μL reaction mixture containing 0.5 mM ascorbate, 50 mM potassium phosphate buffer (pH 7.0), and AtAPX1 protein using a UV-Visible spectrophotometer (Evolution 300 UV-Vis Spectrophotometer; Thermo Scientific, Worcester, MA, United States) for 3 min. Potassium cyanide (KCN 8 mM) was used as an inhibitor of heme-containing protein.

### Molecular Chaperone Activity Assay

The molecular chaperone activity assay was performed as previously described (Lee et al., 2015) by analyzing the ability of AtAPX1 in preventing the thermal aggregation of a heat-sensitive substance, malate dehydrogenase (MDH) (Lee et al., 1997; Moon et al., 2005). MDH was incubated in a 50 mM HEPES buffer (pH 8.0) at 43°C with various concentrations of AtAPX1 or its fractions separated by SEC. The thermal aggregation of the substrate was estimated by monitoring the change in turbidity at 340 nm for 15 min using an Evolution 300 Spectrophotometer (Thermo Scientific) equipped with a thermostatic cell holder.

### Plant Growth Conditions and Stress Treatment

*Arabidopsis thaliana* (Columbia-0 ecotype) seeds were imbibed in sterile water for 30 min at 25°C and then surface sterilized with 4% sodium hypochlorite solution for 10 min, rinsed 5 times with sterile water, plated on one-half strength Murashige and Skoog (1/2 MS) medium supplemented with 1% sucrose and 0.8% phytoagar, and then stratified at 4°C for 3 days. Plants were grown under a 16 h light/8 h dark cycle at 23°C. Then, 10 and 14 days old seedlings were used for abiotic stress treatments. For heat stress, 10 days old seedlings were transferred to a plant growth incubator set at 42°C for 1 h and then transferred to 23°C for recovery for 1 and 3 days. Likewise, 14 days old plants were transferred to 1/2 MS media supplemented with 200 mM NaCl for 6 h. After 6 h, half of the plants were transferred to 1/2 MS media for recovery and samples were collected after 1 day.

### Extraction of Total Plant Protein and Western Blotting

*Arabidopsis* plants were immediately frozen upon harvesting and ground to a fine powder in liquid nitrogen using a pestle and mortar. A total of 0.5 mL of protein extraction buffer (50 mM Tris–HCl pH 7.5, 10% glycerol, 150 mM NaCl, 10 mM MgCl_2_, 5 mM EDTA, 5 mM DTT, and 1 × protease inhibitors) was added to each sample and incubated at 4°C for 1 h with constant rotating. The homogenate was centrifuged at least two times at 13,000 × *g* for 10 min and the supernatant was collected after each centrifugation. The protein content was measured using the Pierce BCA protein assay kit (Thermo-Scientific). Western blot analysis of total protein run on a 10% native-PAGE was performed to investigate the conformational changes in the AtAPX1 protein. Polyclonal mouse anti-AtAPX1 antibody was used to detect AtAPX1 protein complexes from total protein extracts. Native-PAGE was performed as previously described (Moon et al., 2005).

### Treatment With GSNO, SIN-1, and NaHS

The recombinant AtAPX1 protein was incubated with various concentrations (0–1 mM) of S-nitrosoglutathione (GSNO) at 25°C for 30 min in the dark to achieve S-nitrosylation of protein. Excess of GSNO was removed using Amicon centrifugal filter units (10 k NMWL) (Merck Millipore). H_2_S precursor sodium hydrogen sulfide (NaHS) was used to induce sulfhydration in the AtAPX1 recombinant protein. The AtAPX1 protein was incubated with various concentrations of freshly prepared NaHS (10 nM–100 μM) at 4°C for 30 min in the dark. Residual NaHS was removed using Amicon centrifugal filter units (10 k NMWL) (Merck Millipore). The S-nitrosylated and S-sulfhydrated proteins were detected with anti-biotin HRP linked antibody (Cell Signaling Technology, Seoul, Korea) using a biotin switch assay with slight modifications (Jaffrey and Snyder, 2001; Mustafa et al., 2009). The SIN-1 molecule (3-morpholinosydnonimine), a protein nitrating compound, was used as a peroxynitrite donor. Recombinant AtAPX1 protein was incubated with various concentrations (0–10 mM) of freshly prepared SIN-1 at 37°C for 1 h in the dark. Tyrosine nitrated proteins were detected with 3-nitrotyrosine antibody (Invitrogen, Rockford, IL, United States). Treated protein was used for the APX enzymatic activity assay, chaperone activity assay, SDS-PAGE, and native-PAGE analysis.

## Results

### AtAPX1 Exists in Different Forms of Varying Sizes

**FIGURE 1:**
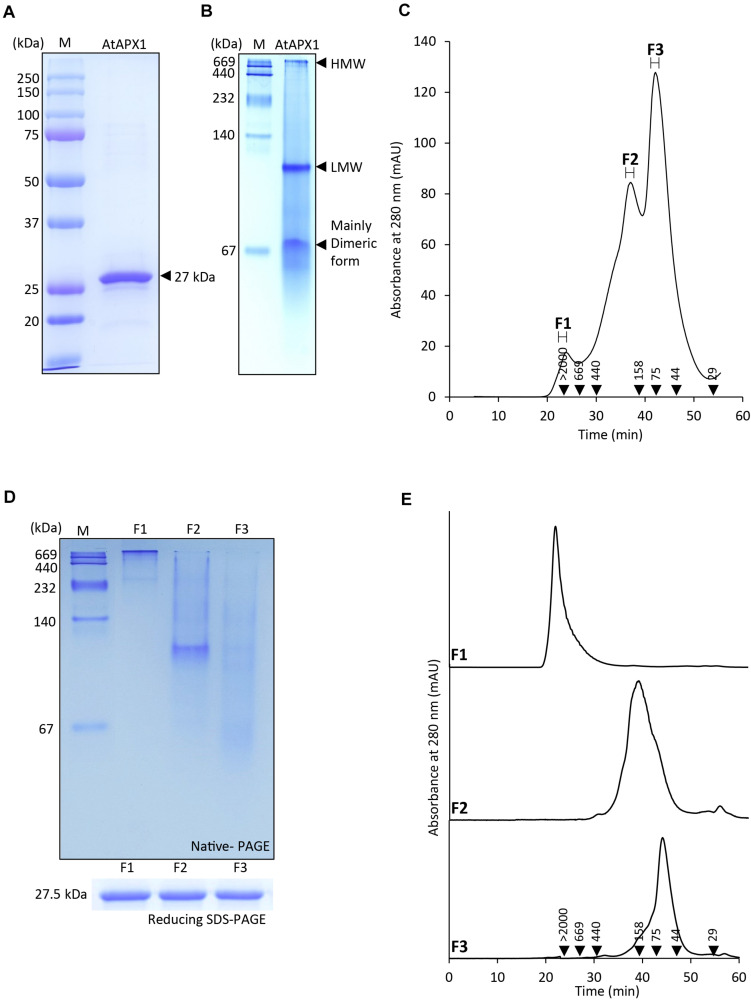
Protein structure of AtAPX1 protein analyzed by **(A)** reducing SDS-PAGE, **(B)** native-PAGE, and **(C,E)** size exclusion chromatography (SEC). The values in the chromatogram represent the molecular weights of the standard proteins: blue dextran (>2,000 kDa), thyroglobulin (669 kDa), ferritin (440 kDa), aldolase (158 kDa), conalbumin (75 kDa), ovalbumin (44 kDa), and carbonic anhydrase (29 kDa). The three peaks (F1-F3) of separated proteins were collected and used for further analysis. **(D)** The fractions were separated by 10% native-PAGE and 12% reducing SDS-PAGE. **(E)** Each of the fractions was rechromatographed by SEC to confirm their stability.

In order to understand the structural status of the AtAPX1 protein, we used recombinant purified protein for analysis by SDS-PAGE, native-PAGE, and SEC. Figure 1A shows that recombinant AtAPX1 protein existed largely as a single band at a size of 27 kDa in 12% SDS-PAGE in the presence of DTT (reducing). Native-PAGE analysis revealed the presence of a dimeric form besides the low-molecular weight (LMW) and high-molecular weight (HMW) complexes of the AtAPX1 protein (Figure 1B). To further investigate the oligomeric status, we analyzed the AtAPX1 protein using size exclusion chromatography (SEC) (Figure 1C). SEC analysis showed three different peaks for the APX1 protein, which were represented by F1, having HMW complexes, the F2-LMW form, and the predominant F3 peak representing the major dimeric form. Each of the fractions corresponding to these three peaks were collected and reanalyzed using SEC to check their stability (Figure 1E). Collected fractions were subjected to reducing 12% SDS-PAGE, which demonstrated the presence of a single band at 27 kDa, representing the monomeric unit of AtAPX1, in all fractions. However, 10% native-PAGE analysis of collected fractions (Figure 1D) indicated that proteins and complexes in the F1 fraction (F1 > 2,000 kDa) stuck on the top of the 10% native-PAGE and were not able to travel through the gel because of their higher molecular weight. We found that the second SEC fraction (F2) showed a band of proteins between 158 and 440 kDa, whereas the F3 fraction corresponding to the dimeric form of AtAPX1 appeared below 158 kDa, representing the major peak in SEC analysis. The slight variation observed in the size of fractions in native-PAGE and SEC results might be due to the difference in underlying principle of both the separation techniques. These results indicated that AtAPX1 existed in multimeric forms, with the dimeric form being the dominant one.

### Dual Function of APX as a Peroxidase and a Chaperone

**FIGURE 2:**
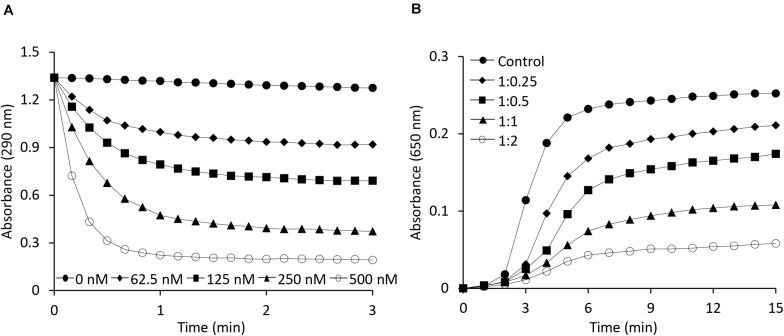
Functional analysis of AtAPX1 protein as ascorbate peroxidase and molecular chaperone. **(A)** The APX activity was monitored by measuring the reduction in absorbance at 290 nm using (◆) 62.5 nM, (◼) 125 nM, (▲) 250 nM, (○) 500 nM, and (●) 0 nM purified AtAPX1 protein. **(B)** Chaperone activity was measured at 650 nm using malate dehydrogenase (MDH) as a substrate. Thermal aggregation of 1 μM MDH was examined at 43°C for 15 min in the presence of purified AtAPX1 protein in molar ratios of MDH:AtAPX1, (◆) 1:0.25, (◼) 1:0.5, (▲) 1:1, and (○) 1:2. (●) Control indicated the thermal aggregation of MDH in the absence of AtAPX1.

Both OsAPX2 (Hong et al., 2018) and 2-Cys Prx (Jang et al., 2004), which exists in multimeric forms, are known to play a dual role, serving both as a peroxidase and a chaperone. The ascorbate-glutathione cycle is a pivotal antioxidant system involved in the regulation of H_2_O_2_ levels (Noctor and Foyer, 1998). Ascorbate peroxidase, being an important enzymatic antioxidant of this cycle, catalyzes the reduction of H_2_O_2_ to water using ascorbate as a specific electron donor. To investigate the role of the AtAPX1 recombinant protein as a typical ascorbate peroxidase, we performed an APX enzymatic activity assay by measuring the decrease in absorbance at 290 nm due to the oxidation of ascorbate. We accordingly observed that AtAPX1 showed an increase in enzymatic activity in a dose-dependent manner, as shown in Figure 2A.

To identify the potential function of AtAPX1 as a molecular chaperone, we investigated the chaperone activity of the recombinant protein by assessing its ability to inhibit the thermal aggregation of malate dehydrogenase (MDH), a heat-sensitive substrate. We found that the AtAPX1 protein showed a high chaperone activity as incubation of MDH with increasing amounts of AtAPX1 resulted in a concomitant decrease in the aggregation of MDH at 43°C (Figure 2B). We further noted that the aggregation of MDH was effectively suppressed at a subunit molar ratio of MDH to AtAPX1 of 1:2. Therefore, our results suggested that AtAPX1 exhibits both peroxidase and chaperone activities, similar to OsAPX2.

### Functional and Structural Status of AtAPX1 Are Interconnected

**FIGURE 3:**
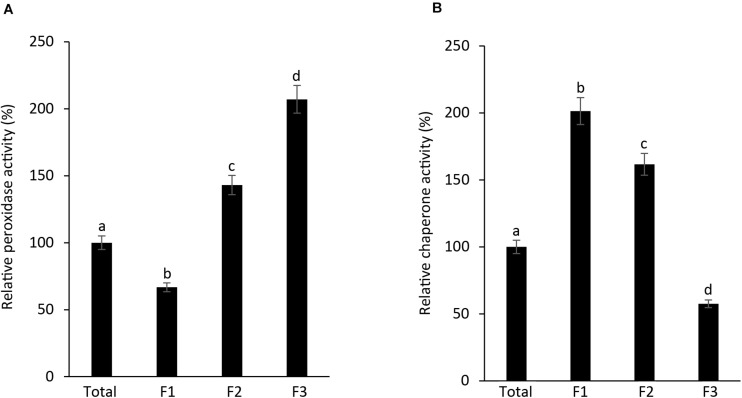
AtAPX1 functions are related to differently sized protein structures. We separated the AtAPX1 protein into three fractions corresponding to the HMW complexes (F1), the LMW form (F2), and the dimeric form (F3). **(A)** Peroxidase activity of fractions was assayed by monitoring the reduction of ascorbate at 290 nm. **(B)** Chaperone activity was evaluated using a 1:1 molar ratio of MDH and AtAPX1. The activities of the three AtAPX1 protein fractions were compared with those of total protein which was set to 100%. Data are means (±) standard deviation of three independent experiments. Different letters indicate significant differences at *P* < 0.05 between the total protein and fractions by one-way ANOVA with Tukey’s test.

As AtAPX1 appears in multimeric forms ranging from LMW to HMW form, with dual function, acting both as a peroxidase and a molecular chaperone, we investigated the connection between its structural conformations and functions. We collected and used the fractions (F1-F3) corresponding to the peaks of SEC for functional analysis. We found that the highest APX activity was exhibited by F3, the lowest molecular weight fraction, whereas the lowest APX activity was exhibited by F1, the highest molecular weight fraction. Furthermore, we observed that the APX activity of the F1 fraction was lower than that of the total protein (Figure 3A). In contrast, the chaperone activity was demonstrated to be the highest in the F1 fraction, which represents high molecular weight complexes, whereas it was the lowest in the F3 fraction. These results suggested that multimerization of protein subunits promoted the molecular chaperone activity, whereas the LMW form has enhanced the peroxidase activity. Therefore, we concluded that the dual function of AtAPX1 was associated with its ability to form different protein complexes.

**FIGURE 4:**
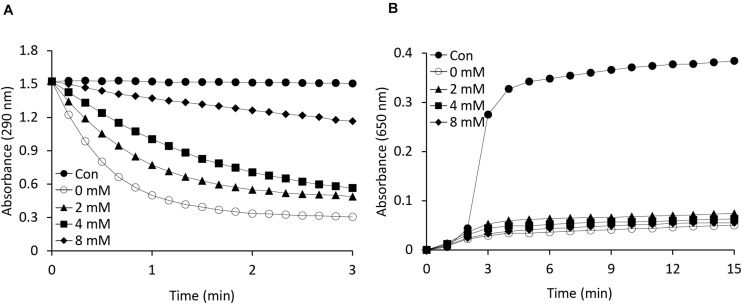
Effects of a heme inhibitor (potassium cyanide) on the **(A)** peroxidase and **(B)** chaperone activities of AtAPX1 protein. To inhibit heme, we incubated AtAPX1 protein with (○) 0 mM, (▲) 2 mM, (◼) 4 mM, and (◆) 8 mM potassium cyanide at 4°C for 15 min. (●) Control indicated measuring the activity without AtAPX1 protein.

As is known, APX is a heme-containing enzyme and its activity is inhibited by potassium cyanide (KCN) and sodium azide, both of which are heme inhibitors (Nakano and Asada, 1981). Heme inhibitors bind to the heme moiety of the APX protein, inhibiting its peroxidase activity. In this study, we also investigated whether the function of APX as a molecular chaperone is being affected by heme inhibitor. To identify any relationship between the APX and chaperone activity of the protein, we performed a potassium cyanide inhibition test by incubating AtAPX1 proteins with various concentrations of KCN for 30 min (Figure 4). We respectively found that the chaperone activity of the AtAPX1 protein was not affected by KCN, whereas its APX activity was effectively suppressed. More specifically, the chaperone activity of KCN-treated AtAPX1 was demonstrated to be similar to that of non-treated protein, suggesting that the chaperone function of AtAPX1 does not depend on the heme moiety or its APX activity.

### Abiotic Stress Modulates Structural Changes in AtAPX1 Protein

**FIGURE 5:**
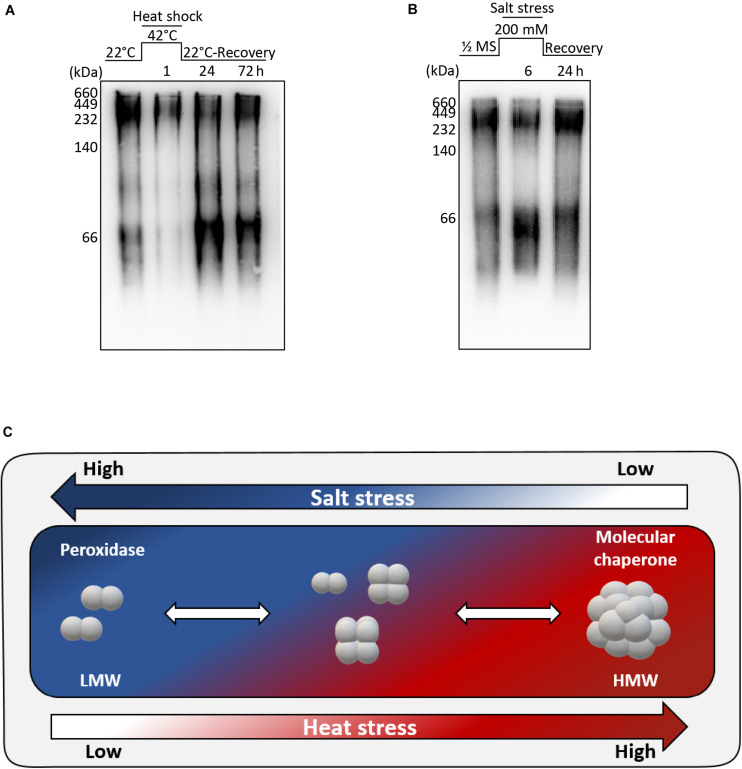
Structural switch in AtAPX1 protein in response to abiotic stress. **(A)** For heat stress, 10 days old seedlings were subjected to heat stress at 42°C for 1 h, and then recovered in a growth chamber at 22°C for 24 h and 72 h. **(B)** For salt stress, 14 days old seedlings were transferred to 1/2 MS supplemented with 200 mM NaCl for 6 h. For recovery, *Arabidopsis* seedlings treated with salt stress were transferred to a new 1/2 MS containing no salt and grown for 24 h. **(A,B)** 20 μg of total protein was separated on 10% native-PAGE, followed by western blot analysis with an anti-AtAPX1 antibody (1:2,500 dilution). M, marker. **(C)** Model representation of molecular switching of AtAPX1 in response to abiotic stresses. APX1 in *Arabidopsis* exists in discretely sized multiple structures with a diverse range of molecular states, including dimeric, LMW, and HMW structures. AtAPX1 protein is converted to dimer or LMW forms under salt stress, whereas it is mainly present in HMW form under heat stress. This reversible switching is accompanied by the change of enzymatic functions of AtAPX1 between peroxidase and molecular chaperone.

We found that the recombinant AtAPX1 protein showed oligomeric forms besides the major dimeric form and these different forms appear to play varying roles depending on the structural status of the protein. The yeast 2-Cys Prx was reported to shift from a LMW form to a HMW form after heat and oxidative stress, accompanied by a functional switching from peroxidase to molecular chaperone (Jang et al., 2004). In order to find out the effects of abiotic stress on the structural status of the AtAPX1 protein, we conducted a number of *in vivo* experiments. Col-0 plants were grown for 10 days and then transferred to a growth chamber set at 42°C for 1 h. For recovery, plants were transferred back to normal conditions and samples were collected after 1 and 3 days. Total protein was extracted and used for western blotting after native-PAGE. We noted that protein from control samples existed in different oligomeric forms similar to recombinant AtAPX1 proteins. Most of the protein was in HMW complexes, with the remaining existing in oligomeric and LMW complexes (Figure 5A). We also observed that after 1 h of heat stress, most of the protein formed HMW complexes; however, when plants were kept under recovery conditions for 3 d, the protein reverted back to its original form.

For the salt stress, 14 days old seedlings were transferred to 1/2 MS supplemented with 200 mM NaCl for 6 h. One half of the samples were collected, and the other half was transferred to 1/2 MS for recovery and samples were collected after 1 day. We found that the AtAPX1 protein from control samples detected using an anti-AtAPX1 antibody exhibited the same pattern of bands in native-PAGE, as shown in Figure 5A, whereas the majority of protein shifted to the LMW form after salt stress. As expected, this shift to the LMW form was reverted following the recovery of plants (Figure 5B).

We observed that the protein exhibited a transition from dimeric units to HMW complexes under heat stress, whereas the HMW complexes were dissociated under salt stress (Figure 5C). These results indicated that abiotic stress modulates the structural status of AtAPX1 *in vivo.*

### Functional and Conformational Changes of AtAPX1 Induced by NO

**FIGURE 6:**
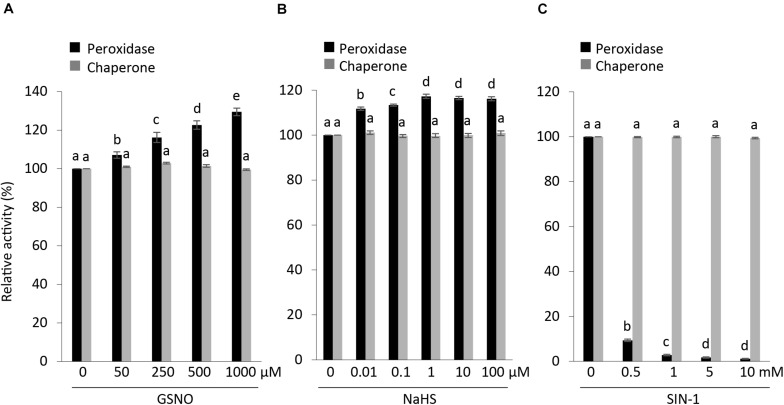
Effects of posttranslational modifications on structural and functional status of AtAPX1. Comparison of peroxidase and chaperone activity of AtAPX1 protein treated with **(A)** GSNO (50–1,000 μM) for 30 min at 25°C in dark (S-nitrosylation), **(B)** NaHS (0.01–100 μM) for 30 min at 4°C in dark (S-sulfhydration), and **(C)** SIN-1 (0.5–10 mM) for 1 h at 37°C in dark (tyrosine nitration). The activities of treated protein were compared to non-treated protein whose activity was set to 100%. The black bars represent peroxidase activity and gray bars represent chaperone activity. Data are means (±) standard deviation of three independent experiments. Different letters indicate significant differences at *P* < 0.05 between non-treated and treated protein samples by one-way ANOVA with Tukey’s test.

S-nitrosylation, which binds a NO group to cysteine residues of proteins, thus modifying their function, is one of the most common posttranslational modifications (Astier et al., 2012; Begara-Morales et al., 2014). In particular, GSNO is known to regulate the activity of APX1 either positively or negatively (De Pinto et al., 2013; Begara-Morales et al., 2014; Yang et al., 2015). We checked the effects of GSNO on the peroxidase and chaperone activity along with structural status of APX1. Our results were in accordance with previous reports indicating that S-nitrosylated APX1 showed substantial increase in the APX activity following treatment with GSNO (Yang et al., 2015); however, no effect was observed on its chaperone activity (Figure 6A). Moreover, we noted that native-PAGE analysis indicated that both treated and non-treated AtAPX1 protein samples showed similar structural behaviors (Supplementary Figure 1). In this study, we revealed for the first time that the GSNO-induced change in the activity of APX1 was not related to its oligomeric status.

We also pretreated purified recombinant APX1 protein with (10 nM–100 μM) NaHS for 30 min at 4°C to increase the concentration of *S*-sulfhydrated proteins. Then, we exchanged the buffer to eliminate residual NaHS before performing the modified biotin switch assay and enzymatic activity assays. We accordingly observed an increase in peroxidase activity in NaHS-treated samples, whereas no effect was observed on chaperone activity and the oligomeric status of the AtAPX1 protein (Figure 6B and Supplementary Figure 2).

In order to evaluate the effect of SIN-1 (peroxynitrite donor) on the structure and function of AtAPX1, we incubated APX1 recombinant protein with 0.5–10 mM SIN-1. According to previous reports, SIN-1 inhibited the APX activity in pea plants (Begara-Morales et al., 2014). Our results corroborated with previous reports, as shown in Figure 6C, which depicts the inhibitory effects of the peroxynitrite donor. In contrast to the negative regulation of the peroxidase activity of the SIN-1-treated protein, we did not observe any effects on its chaperone activity. Treatment with SIN-1 did not cause any conformational changes either, as shown in Supplementary Figure 3, indicating that tyrosine nitration did not affect the structural status of AtAPX1.

Our analysis of the functional and structural status of AtAPX1 after posttranslational modifications revealed that PTMs affect the peroxidase activity of AtAPX1 both positively, as in the case of S-nitrosylation and S-sulfhydration, and negatively as in the case of tyrosine nitration. However, PTMs did not affect the chaperone activity in either case. In addition, almost no changes were observed in the structural status of AtAPX1 after PTMs.

## Discussion

Ascorbate peroxidase along with various enzymes and non-enzymatic antioxidants, such as ascorbate, and glutathione constitute the ascorbate-glutathione cycle. This cycle is important for maintaining and regulating the intracellular levels of hydrogen peroxide (Asada, 1992; Noctor and Foyer, 1998). In particular, APX being one of the most important enzymes in the defense system of plants, detoxifies H_2_O_2_ into water using ascorbate as an electron donor (Foyer and Noctor, 2005). The expression of *APX1*, which increases many folds during stress, can be detected in plant tissues even in the absence of stress (Pnueli et al., 2003). Despite being a cytosolic enzyme, it protects chloroplasts against ROS, indicating the importance of cytosolic enzymes (Davletova et al., 2005).

Recently, our group revealed a new function of the cytosolic OsAPX2 from rice, acting as a molecular chaperone besides its regular APX function (Hong et al., 2018). Therefore, to find out whether APX has a dual function not only in rice but also in other plants, we studied the role of the APX protein in *Arabidopsis thaliana*, a model plant. This study demonstrated that APX1 from *Arabidopsis* also possessed a dual function, acting both as a peroxidase and a molecular chaperone (Figure 2). In addition, we confirmed that APX1 existed in forms of various sizes, with these variations in protein conformation being related to its dual function (Figures 1–3). Our results from native-PAGE and SEC analysis (Figure 1) showed that AtAPX1 existed in homo-oligomeric forms, as the recombinant AtAPX1 protein was separated into three different fractions (F1, F2, and F3). We found that among them, F3, which contained mainly the dimeric form showed the highest APX activity, whereas the F1 fraction that contained HMW complexes showed the highest chaperone activity (Figure 3). Previous studies have shown that various proteins, such as OsAPX2 (Hong et al., 2018) and 2-Cys Prxs existing in bacteria (An et al., 2011), yeast (Jang et al., 2004), plant (König et al., 2002; Lee et al., 2015), and human (Moon et al., 2005) have a dual function, serving both as a molecular chaperone and a peroxidase. The functional switching of these proteins between peroxidase and molecular chaperone was correlated with the changes in the oligomeric status of the protein between its LMW and HMW form. These results corroborated with previous studies reporting on low molecular weight forms exhibiting high peroxidase activity, whereas oligomerization promoted a high chaperone activity. Therefore, these results indicated that the dual function of the APX1 protein was closely associated with the changes in its structural conformation.

As is known, APX is a heme-containing enzyme with protoporphyrin as a prosthetic group and its activity is inhibited by various inhibitors, such as sodium azide, potassium cyanide, and other thiol inhibitors (Shigeoka et al., 1980; Chen and Asada, 1990), which are potent inhibitors of heme-containing proteins. To find out whether the heme moiety is involved in the dual function of AtAPX, we measured both of its activities in potassium cyanide-treated AtAPX protein, where the heme moiety has been inactivated. We found that the peroxidase activity was decreased in a concentration-dependent manner, whereas the chaperone activity was not affected (Figure 4). These results excluded the involvement of the heme moiety in the chaperone activity of APX1, suggesting that the heme moiety is important for the peroxidase activity but not for the chaperone activity and conformational changes of APX1.

It has been reported that APX plays an important role in combating various abiotic stresses (Kubo et al., 1995; Karpinski et al., 1997; Koussevitzky et al., 2008). We thus monitored the *in vivo* structural status of AtAPX1 under abiotic stresses and found that it could be regulated by both heat and salt stress (Figure 5). The most significant finding was that the APX protein, which exists in various sizes *in vivo*, transitioned to HMW complexes under heat stress but reverted to various-sized forms during recovery (Figure 5A). Unlike heat stress, in plants subjected to salt stress the APX protein switched to an LMW form, but this switch was reverted when the salt stress was withdrawn (recovery) (Figure 5B). The switch to LMW forms indicated a high peroxidase activity, whereas the transition to HMW complexes represented a high chaperone activity. These *in vivo* results demonstrated the regulation of the dual function and structural status of the AtAPX1 protein by abiotic stresses. Hong et al. (2018) reported that the *in vivo* structural status of OsAPX2 in the IR-29 salt-sensitive rice cultivar was mainly represented by HMW complexes with very few LMW proteins. Therefore, they could not observe the structural change of OsAPX2 from LMW to HMW in response to heat stress *in vivo.* In the present study, we revealed that *Arabidopsis* AtAPX1 existed in various sizes from dimeric to HMW complexes, with their structure being regulated by salt and heat stresses.

It was recently reported that S-nitrosylation, tyrosine nitration, and S-sulfhydration are posttranslational modifications induced by reactive nitrogen species (RNS) and hydrogen sulfide (H_2_S), an endogenous gaseous mediator that can regulate the peroxidase activity of the APX protein either negatively or positively, depending on the plant species (Begara-Morales et al., 2016; Aroca et al., 2018). To date, PTM studies on the APX protein have been limited only to its peroxidase activity. However, as this study identified and confirmed the dual function of APX, we examined the effects of PTMs on the structure and the dual function of APX using enzyme assays, as well as SDS- and native-PAGE. S-nitrosylation and S-sulfhydration are two PTMs targeting cysteine (Cys) residues in proteins. S-nitrosylation is the covalent attachment of a nitric oxide group (-NO) to the thiol group of a Cys residue, forming S-nitrosothiol (SNO), whereas S-sulfhydration involves the formation of a hydropersulfide moiety (-SSH) from Cys (Mustafa et al., 2009). The APX1 protein from *Arabidopsis* has 5 cysteine residues, of which 2 cysteine residues (Cys-32 and Cys-49) are S-nitrosylated (Yang et al., 2015). Cys-32 was also shown to be the target residue for S-sulfhydration when recombinant AtAPX1 protein was treated with NaHS and analyzed by LC-MS/MS (Aroca et al., 2015). Cys-32 is near the propionate side chain of the heme group and hence any modifications, such as S-nitrosylation and oxidation can cause local conformational changes around the heme group (Sharp et al., 2004; Yang et al., 2015). This situation could directly or indirectly regulate the binding affinity of APX1 with ascorbate, resulting in increased peroxidase activity. In contrast to the positive effects of S-nitrosylation and S-sulfhydration on the peroxidase activity of AtAPX1, the chaperone activity was not affected.

Tyrosine nitration is the addition of a nitro (-NO_2_) group to the tyrosine residue of the target protein, which can promote conformational changes that can lead to gain, loss, or no change in the function of the target protein (Kolbert et al., 2017). Tyrosine nitration induces structural changes by enhancing the hydrophobicity of tyrosine residues (Souza et al., 2008). The pea APX1 was shown to have two of its tyrosines nitrated, of which Tyr235 has been suggested to be the most eligible candidate for inhibiting the activity of APX as it is present close to the pocket of the catalytic center and 3.6 Å away from the heme group (Patterson et al., 1995; Mandelman et al., 1998; Begara-Morales et al., 2014). Treatment of recombinant AtAPX1 with SIN-1 was demonstrated to lead to loss of peroxidase activity in a concentration-dependent manner. Loss of activity due to tyrosine nitration has been observed for most proteins (Corpas et al., 2013). These results concurred with previous reports on the loss of APX activity following tyrosine nitration of the APX1 protein in pea and tobacco (Clark et al., 2000; Begara-Morales et al., 2014). In contrast, the chaperone activity and the structural conformation were not affected by tyrosine nitration, indicating the direct effects of tyrosine nitration on the heme group.

In this work, we studied the link of multimeric forms of the APX1 protein from *Arabidopsis* with its dual function as a peroxidase and a molecular chaperone, and investigated its regulation *in vivo* under abiotic stresses. Moreover, this work was a comprehensive study on the effects of various PTMs on the dual function of AtAPX1, as well as their interconnection with the structural conformations of the protein.

## Data Availability Statement

The original contributions presented in the study are included in the article/Supplementary Material, further inquiries can be directed to the corresponding author/s.

## Author Contributions

SK and SSL conceived and designed the study. SK, PP, D-HB, SH, CC, and M-SC performed the experiments. SK, PP, D-HB, J-HK, SL, and SSL analyzed the data. J-HK, SL, H-WB, SYL, BC, and SSL contributed input and critically reviewed the manuscript. SK and SSL wrote the manuscript. SSL supervised the work. All authors contributed to the article and approved the submitted version.

## Conflict of Interest

The authors declare that the research was conducted in the absence of any commercial or financial relationships that could be construed as a potential conflict of interest.
