# Supplementary material for: Regulation of Dual Activity of Ascorbate Peroxidase 1 From Arabidopsis thaliana by Conformational Changes and Posttranslational Modifications
Source: Front Plant Sci. 2021 Jun 14;12:678111. doi: 10.3389/fpls.2021.678111 (PMC8236860; doi:10.3389/fpls.2021.678111)
Supplement: Supplementary file 1 [file Data_Sheet_1.docx]

Supplementary Material

## Supplementary Figures


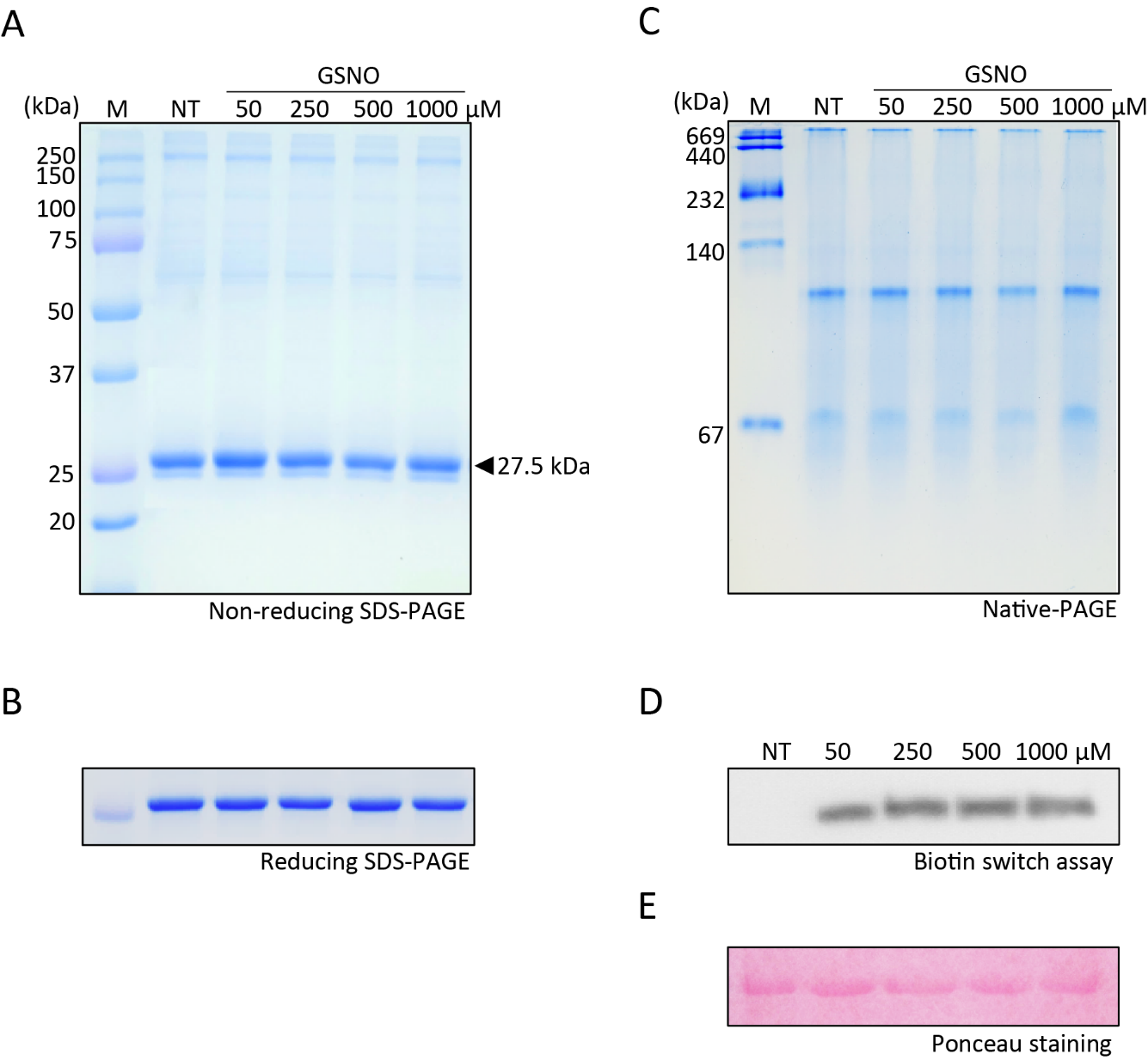


**Supplementary Figure 1.**Structural analyses and detection of S-nitrosylated AtAPX1 protein.AtAPX1 protein treated with various concentrations (50‒1000 µM) of GSNO at 25°C for 30 min in the dark, and then analyzed by **(A)**non-reducing SDS-PAGE, **(B)** reducing SDS-PAGE, and **(C)** native-PAGE.(**D**) S-nitrosylated proteins were detected with biotin switch method. Detection of biotinylated AtAPX1 was achieved using an anti-biotin antibody. **(E)** PonceauS staining was used as loading control.


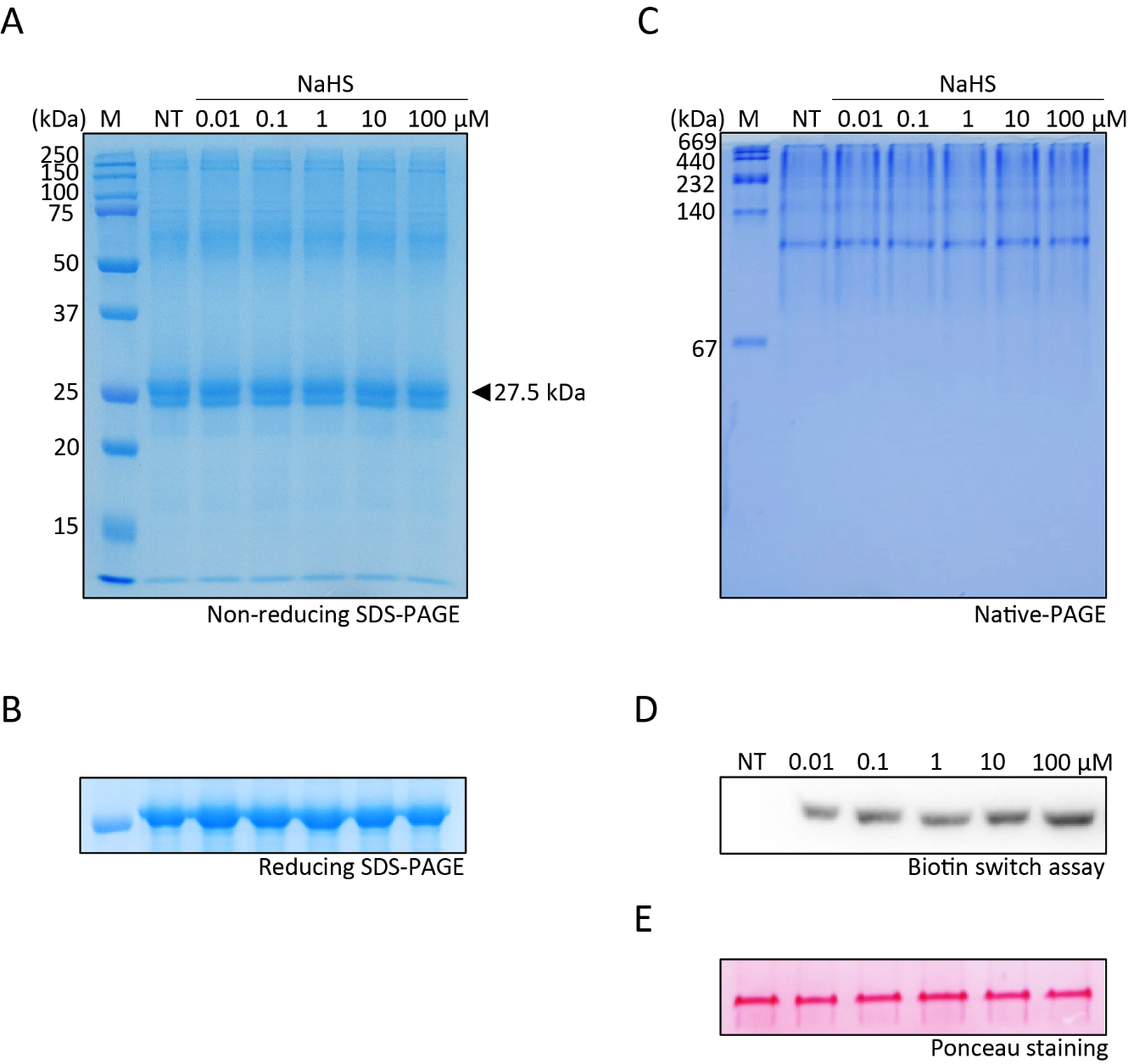


**Supplementary Figure 2.**Structural analyses and detectionof S-sulfhydrated AtAPX1 protein. AtAPX1 protein treated with various concentrations (0.01‒100 µM) of NaHSat 4°C for 30 min in the dark, and then analyzed by **(A)** non-reducing SDS-PAGE, **(B)** reducing SDS-PAGE, and **(C)** native-PAGE. (**D**) S-sulfhydrated proteins were detected with modified biotin switch method. Detection of biotinylated AtAPX1 was achieved using an anti-biotin antibody. **(E)** PonceauS staining was used as loading control.


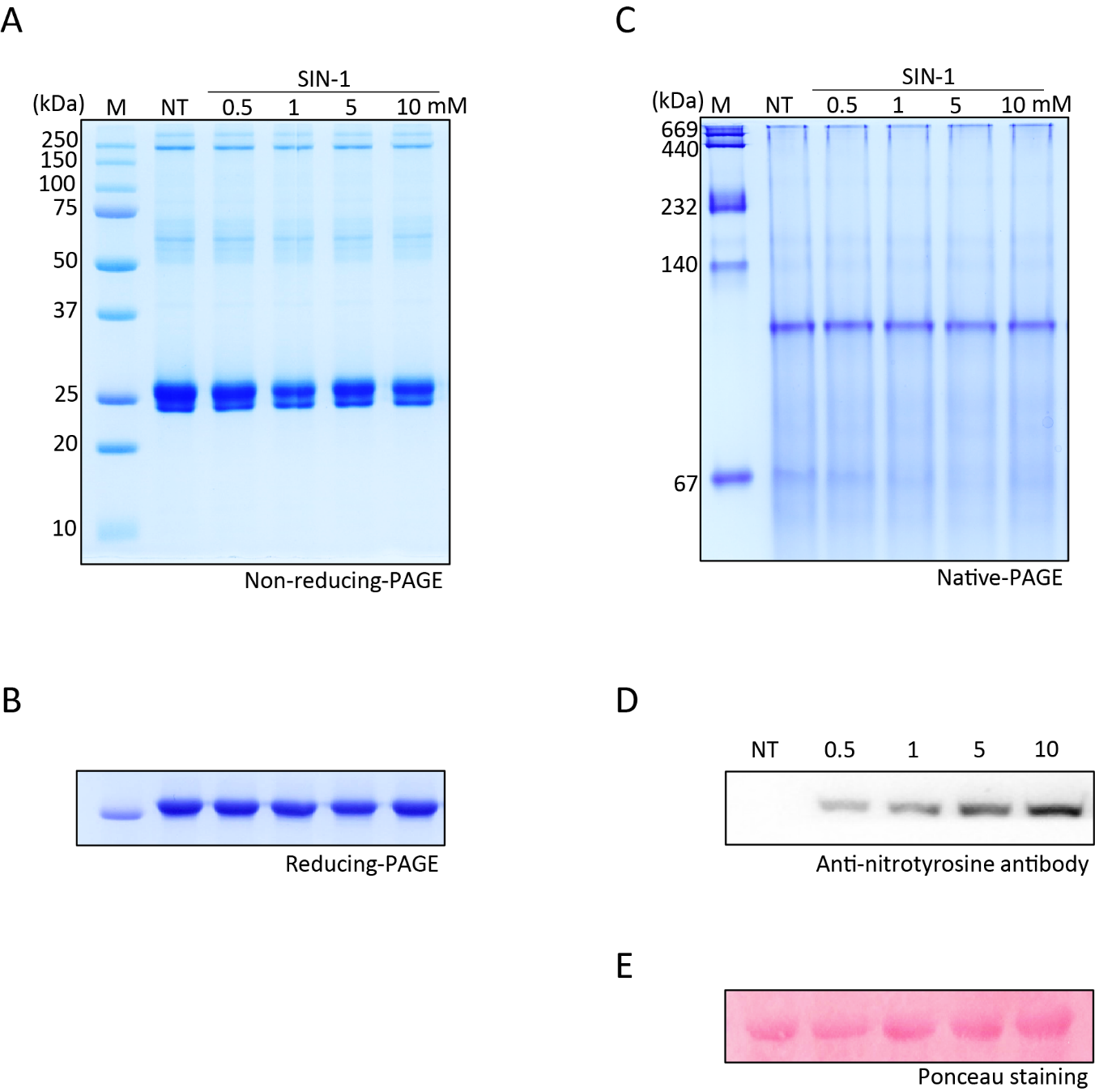


**Supplementary Figure 3.**Structural analyses and detectionof tyrosine nitrated AtAPX1 protein. AtAPX1 protein treated with various concentrations (0.5‒10 mM) of *SIN-1* (peroxynitrite donor) at 37 °C for 1 h in the dark, and then analyzed by **(A)** non-reducing SDS-PAGE, **(B)** reducing SDS-PAGE, and **(C)** native-PAGE. (**D**) Tyrosine nitrated protein were detected with anti-nitrotyrosine antibody (1:5000 dilution). (**E)** PonceauS staining was used as loading control.
